# 

**Published:** 1863-12

**Authors:** 


### CONTENTS OF THE DECEMBER NUMBER.

PAGE.

SELECTED.

The Morbid Conditions of the Blood. A Lecture by Austin Flint, M. D. 530

On the Structure and Development of the Vertebrate Skeleton. By Prof. Huxley, F. R. S. ........ 539

Retreat for Intemperate Women ........ 547

ORIGINAL COMMUNICATIONS.

Clininical Lectures on Disea-es of the Eye. By E. L. Holmes, M. D., of Chicago, Lecturer on Diseases of the Eye and Ear, in Rush Medical College, etc ........ 548

Pyæmia, or Leukæmia. By Geo. R. Weeks, Surgeon of U. S. Vol., in charge of Church Hospital ........ 554

EDITORIAL AND MISCELLANEOUS ........ 561

**E. L. HOLMES, M. D.,**

Lecturer on Diseases of the Eye and Ear.

